# Maternal and Neonatal Immune Signatures at Birth and Future Type 1 Diabetes: Potential Antiviral and Inflammatory Immune Profiles

**DOI:** 10.3390/cells15212036

**Published:** 2026-11-09

**Authors:** Shamila D. Alipoor, Sheida Ardeshirilajimi, Johnny Ludvigsson

**Affiliations:** 1 Division of Pediatrics, Department of Biomedical and Clinical Sciences, Linköping University, 581 83 Linköping, Sweden; shamila.darvish.alipoor@liu.se (S.D.A.); sheida.ardeshirilajimi@liu.se (S.A.); 2 Crown Princess Victoria Children’s Hospital, Linköping University Hospital, 581 85 Linköping, Sweden

**Keywords:** type 1 diabetes, prenatal immune programming, proteomics, DNA methylation, antiviral immunity

## Abstract

Background/Objectives: Prenatal immune programming may contribute to the development of type 1 diabetes (T1D), but the maternal and neonatal immune profiles associated with subsequent disease remain poorly understood. This study aims to investigate maternal and neonatal serum proteomic signatures and cord blood DNA methylation patterns in relation to future T1D. Methods: We analyzed maternal serum proteomic profiles collected at delivery, neonatal cord serum proteomic profiles, and cord blood DNA methylation data from mothers and children in the ABIS birth cohort. Results: After false discovery rate (FDR) correction, 60 maternal proteins, but only 3 neonatal proteins, differed significantly between the T1D and control groups. Both proteomic analyses showed enrichment of immune and inflammatory pathways, with Th1/Th2, Th17, IL-17, and chemokine signaling additionally enriched in neonatal serum. In a further analysis restricted to virus-annotated KEGG pathways, the enriched pathways in both datasets converged on shared immune and inflammatory signaling components and did not indicate infection with a specific pathogen. Network integration identified shared immune hubs, including TNF, CD40LG, and multiple CCL/CXCL chemokines. Cord blood DNA methylation changes were modest, although nominally significant differentially methylated genes (DMGs) were associated with immune-related and host–pathogen interaction pathways, showing biological convergence with the proteomic findings. Conclusion: Maternal and neonatal immune profiles at birth may reflect inflammatory processes associated with subsequent T1D development. Maternal serum proteomics provided stronger statistical evidence, whereas the neonatal findings were hypothesis-generating. Further studies in larger independent cohorts, including targeted protein assays, are needed to validate these findings and clarify their biological significance.

## 1. Introduction

Type 1 diabetes (T1D) is a complex disease characterized by progressive loss of pancreatic β-cell function and insulin dependence, in which autoimmune mechanisms play a central role [1]. The autoimmune process can begin years before clinical diagnosis [2,3] and is shaped by the interplay between genetic background and environmental influences acting from fetal life onward [4]. In addition to immune dysregulation, non-autoimmune mechanisms, including β-cell stress [5] and metabolic disturbances [6], may contribute to progression toward clinical disease, particularly during periods of rapid growth [7], infection, or increased insulin resistance [8].

Evidence from birth and early-childhood studies suggests that early-life factors may influence the subsequent development of islet autoimmunity and T1D [9]. These findings raise the possibility that prenatal exposures shape immune programming during a critical early window of T1D pathogenesis. Understanding the biological signals present during this period may therefore provide insight into how environmental influences interact with genetic susceptibility before disease becomes clinically apparent.

A viral contribution to T1D has long been proposed, and several viruses have been implicated. Enteroviruses, particularly coxsackievirus B, have been studied most extensively [10,11,12,13], but no single virus has been consistently identified as the causal trigger [10,11]. Epidemiological studies have reported associations between maternal infection during pregnancy, including enterovirus infection, and increased T1D risk in offspring. However, the effect sizes are modest and vary according to gestational timing and the age at disease onset [14,15,16,17]. Furthermore, low-grade enteroviral infection, including viral protein and RNA in β cells, has been detected in pancreatic islets from individuals with newly diagnosed T1D [18,19]. Together, these findings support the possibility that infection-related immune activation during gestation or early life, rather than a single specific virus, may contribute to later T1D susceptibility. Such effects may not depend solely on persistent viral infection but could also reflect immune programming during critical periods of fetal and early-life immune maturation.

Maternal health and immune status during pregnancy may influence offspring immune development through several mechanisms, including transplacental transfer of antibodies and soluble immune mediators, as well as effects on fetal hematopoiesis and T-cell differentiation [20]. Immune pathways commonly activated during antiviral responses, including type I interferon signaling, TNF/NF-κB activation, and chemokine-mediated leukocyte recruitment, also contribute to β-cell-directed autoimmunity [21]. Because these pathways are shared across many viral infections, circulating immune proteins may capture antiviral or inflammatory immune activation regardless of the specific pathogen.

Despite this biological plausibility, maternal–fetal immune associations at birth in relation to future T1D have not been systematically characterized. Most previous studies have focused on a single compartment, such as cord blood or peripheral blood from children, or on a single molecular layer, such as DNA methylation or transcriptomics [9]. Few prospective studies have profiled both maternal and neonatal immune proteins at delivery, and it remains unclear whether prenatal exposures are associated with coordinated maternal–neonatal immune signatures at birth.

In the present study, we address this gap by integrating maternal serum proteomic profiles obtained at delivery, neonatal serum proteomic profiles obtained at birth, and exploratory cord blood DNA methylation data. We aimed to explore maternal and neonatal immune signatures at birth in relation to T1D development and to investigate the biological pathways represented by these signatures. This exploratory analysis may provide insights into early-life immune patterns associated with future T1D and generate hypotheses for future studies investigating molecular markers for early risk assessment and preventive strategies.

## 2. Materials and Methods

### 2.1. Data Source and Study Design

This study used data from two independent groups within the ABIS prospective birth cohort [22,23]. The first group (A) comprised children for whom both cord blood DNA methylation and neonatal serum proteomic data were available and was used to investigate molecular alterations associated with the future development of T1D. This group included 32 children who later developed T1D and 48 controls with no recorded diagnosis of autoimmune or immune-mediated disease. The second group (B) consisted of maternal serum samples collected at delivery and was used to identify maternal protein signatures associated with the subsequent development of T1D in the offspring. This group included 24 mothers of children who later developed T1D and 38 control mothers. A sensitivity power analysis was performed to evaluate the study’s ability to detect differences in varying effect sizes given the available sample sizes. The analysis procedure is described in Supplementary File S1.

### 2.2. Ethics

The ABIS study was approved by the Research Ethics Committees of the Faculty of Health Sciences, Linköping University (Ref. 1997/96287 and 2003/03-092) and the Medical Faculty of Lund University (Dnr 99227 and Dnr 99321). Linkage to the Swedish National Patient Register and the National Drug Prescription Register was approved by the Swedish Data Inspection Board (Dnr 03-513 and 2018-318-32). Written informed consent was obtained from all parents or legal guardians. The present secondary analysis was conducted under these ethical approvals.

### 2.3. DNA Methylation Profiling

Genome-wide DNA methylation profiling of cord blood samples was performed as previously described [24]. Briefly, genomic DNA was extracted from cord blood, bisulfite- converted, and analyzed using the Illumina Infinium MethylationEPIC BeadChip (Illumina, San Diego, CA, USA). Raw IDAT files were processed and quality-controlled in R software (version 4.3.0, R Foundation for Statistical Computing, Vienna, Austria) following our previously published pipeline, including normalization, probe filtering, batch correction, and identification of differentially methylated CpG sites (DMCs) using the limma package (version 3.58.1, Walter and Eliza Hall Institute of Medical Research, Melbourne, Australia), with adjustment for sex and estimated cell type proportions. DMCs were annotated to differentially methylated genes (DMGs) using the IlluminaHumanMethylationEPICanno.ilm10b4.hg19 annotation package (version 0.6.0, Kasper Daniel Hansen, Johns Hopkins University, Baltimore, MD, USA). The previously published DMG list [24] was used directly for the integrative analyses in the present study and was not re-derived. The complete list of DMGs used as input is provided in Supplementary Table S2.

### 2.4. Serum Proteomics

Circulating protein levels in neonatal and maternal serum were measured using Olink Proteomics (Olink Proteomics AB, Uppsala, Sweden). Neonatal serum samples were analyzed using the Olink Explore 384 Inflammation and Oncology panels, deployed on the next-generation sequencing-based Olink Explore platform, covering 727 protein assays. Protein abundance was expressed as normalized protein expression (NPX) values on the log_2_ scale according to the manufacturer’s standard quality control and normalization procedure. Maternal serum samples were analyzed using the Olink Reveal platform, covering 1037 protein assays reported as NPX values on the log_2_ scale, following the manufacturer’s quality control procedures and intensity normalization.

Differential protein abundance analyses were performed separately for the maternal and neonatal datasets using platform specific normalized NPX values. No cross-platform normalization was performed, and NPX values were not directly compared quantitatively between maternal and neonatal samples. Duplicate measurements were averaged where applicable, and proportions of missing values were assessed. Principal component analysis (PCA) was performed separately for each dataset using the OlinkAnalyze R package (version 5.1.0, Olink Proteomics AB, Uppsala, Sweden) to identify potential outliers, defined as samples with PC1 or PC2 scores more than 3 standard deviations from the mean. Differential protein abundance was assessed using the limma package in R. *p* values were adjusted for multiple testing using the Benjamini–Hochberg false discovery rate (FDR). To keep the two datasets comparable, proteins with nominal *p* < 0.05 and an absolute ΔNPX ≥ 0.2 were considered differentially abundant and used for downstream analyses. This threshold of 0.2 was used as an exploratory criterion to identify proteins showing differences in abundance between groups. Functional enrichment analyses of differentially abundant proteins were performed [25] using Kyoto Encyclopedia of Genes and Genomes (KEGG) pathways [26].

### 2.5. Integrative Network Analysis

To investigate molecular interactions across the epigenome and proteome, previously identified DMGs from cord blood and differentially abundant serum proteins from the present study were integrated using a network-based approach. Gene and protein symbols were combined and mapped to the STRING database (version 12.0) [27] to construct protein–protein interaction (PPI) networks. Only high-confidence interactions (combined interaction score ≥ 0.7) were retained. Network visualization and downstream topological analyses were performed in R using the STRINGdb (version 2.14.3, Swiss Institute of Bioinformatics, Lausanne, Switzerland), igraph (version 2.2.1, cynkra GmbH, Zurich, Switzerland), and ggraph (version 2.2.2, RStudio/Posit, Boston, MA, USA) packages. Functional interpretation of the resulting networks was based on Gene Ontology (GO) [25] and KEGG [26] enrichment analysis. Network connectivity was further evaluated using permutation testing against 5000 randomly sampled protein sets. Details of the procedure are provided in Supplementary File S2.

### 2.6. Functional Assignment of Immune Proteins to Modules

Maternal and neonatal immune-related proteins were assigned to functional modules based on their primary biological function and KEGG pathway associations. Module categories were defined using established immune pathway classifications and informed by the preceding pathway enrichment analyses. The modules comprised chemokine-mediated recruitment, T-cell activation, cytotoxic/NK-cell responses, TNF/NF-κB signaling, interferon signaling, and vascular/stress response. Proteins associated with multiple pathways were assigned to the module that best represented their primary biological function. The complete protein-to-module assignments, primary biological functions, and associated KEGG pathways are provided in Supplementary Table S5.

## 3. Results

The DMGs identified in our previously published cord blood epigenome-wide analysis of the ABIS cohort were used as the epigenetic component of the present study; the full list of imported DMGs is provided in Supplementary Table S2. These DMGs were integrated with newly generated neonatal and maternal serum proteomic data to investigate molecular interactions associated with the future development of T1D.

For proteomic data, following quality-control filtering, no missing NPX values remained in either the maternal or neonatal dataset; therefore, no imputation was required (Supplementary Table S4). PCA was performed separately for each dataset to assess potential outliers, and no samples were excluded based on the PCA results (Supplementary File S1). Differential abundance analyses identified proteins that differed in neonatal cord serum from children who later developed T1D and in maternal serum collected at delivery, each compared with their respective control groups. After FDR correction, 60 maternal proteins but only 3 neonatal proteins remained significant (FDR < 0.05). The maternal results therefore represent the primary discovery signal in this study, and the neonatal results, which rely mainly on nominal *p* values, are presented as hypothesis-generating. Complete lists of differentially abundant proteins for each comparison are provided in Supplementary Table S3.

To investigate cross-layer molecular interactions, the previously identified DMGs, together with the neonatal and maternal differentially abundant proteins, were integrated using PPI network analysis, enabling a systems-level comparison of fetal epigenetic, neonatal proteomic, and maternal proteomic signatures.

### 3.1. Neonatal Serum Proteomics Suggests an Immune Landscape Enriched for Inflammatory and Infection-Related Pathways in Future T1D

**Figure 1:**
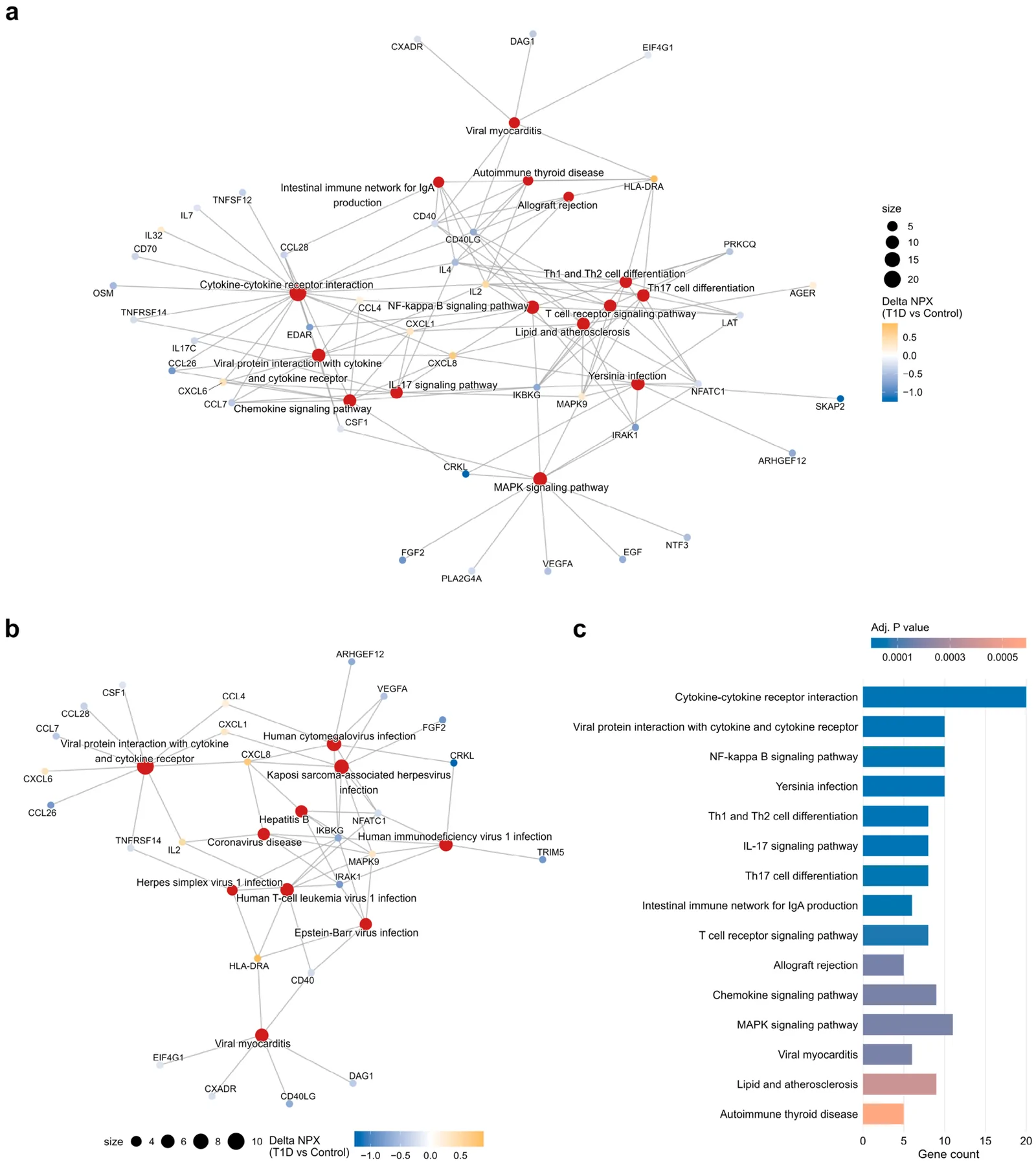
Neonatal serum immune networks and infection-annotated pathways in future T1D. (**a**) Pathway–gene network of neonatal cord serum proteins showing the top enriched KEGG pathways in children who later developed T1D compared with controls. Nodes represent proteins and pathways; edges indicate membership of proteins in specific pathways. Enriched pathways include cytokine–cytokine receptor interaction, NF-κB signaling, Th1/Th2 and Th17 differentiation, IL-17 signaling, T-cell receptor signaling, MAPK signaling, and chemokine signaling. (**b**) Focused neonatal pathway–gene network restricted to KEGG pathways annotated as infection or host–pathogen interaction. Enriched pathways include viral protein interaction with cytokine and cytokine receptor, viral myocarditis, human cytomegalovirus infection, hepatitis B, herpes simplex virus 1 infection, Epstein–Barr virus infection, coronavirus disease, and human T-cell leukemia virus 1 infection. The network shows substantial overlap across multiple virus-associated pathways, converging on a shared set of inflammatory and immune signaling molecules rather than distinct virus-specific signatures. (**c**) Enrichment of the 15 KEGG pathways shown in panel a, ranked by adjusted *p* value; bar length shows the number of proteins in each pathway. Enrichment was based on nominally significant proteins (*p* < 0.05).

In neonatal cord serum, children who later developed T1D showed differences in immune-related proteins compared with controls. KEGG pathway analysis showed enrichment of immune and signaling pathways, including cytokine–cytokine receptor interaction, viral protein interaction with cytokine and cytokine receptor, NF-κB signaling, Th1 and Th2 cell differentiation, Th17 cell differentiation, IL-17 signaling, T-cell receptor signaling, MAPK signaling, and chemokine signaling (Figure 1a,c; Supplementary File S1). Network analysis further suggested that these pathways were linked through a set of shared cytokines, chemokines, and immune regulatory genes, with interleukins, TNF-related molecules, and multiple CCL/CXCL family genes occupying central positions within the network. However, in a permutation test, a STRING protein–protein interaction network built from the neonatal differentially abundant proteins was not more connected than networks built from 5000 random protein sets (*p* = 0.14; Supplementary File S2), which suggests that the connections among these proteins may partly reflect the general connectivity of immune proteins on the Olink panels rather than a T1D-specific pattern.

To further examine infection-related immune signatures, the KEGG enrichment results were restricted to pathways associated with infection and host–pathogen interactions, based on the significant proteins at nominal *p* < 0.05. A compact interaction network emerged, comprising viral protein interaction with cytokine and cytokine receptor, viral myocarditis, human cytomegalovirus infection, hepatitis B, herpes simplex virus 1 infection, Epstein–Barr virus infection, coronavirus disease, and human T-cell leukemia virus 1 infection (Figure 1b). The network showed overlap across multiple virus-associated pathways, converging on a common set of inflammatory and immune signaling molecules (Figure 1b).

### 3.2. Maternal Serum Proteomics at Delivery Reveals an Inflammatory Immune Profile in Mothers of Children with Later T1D

**Figure 2:**
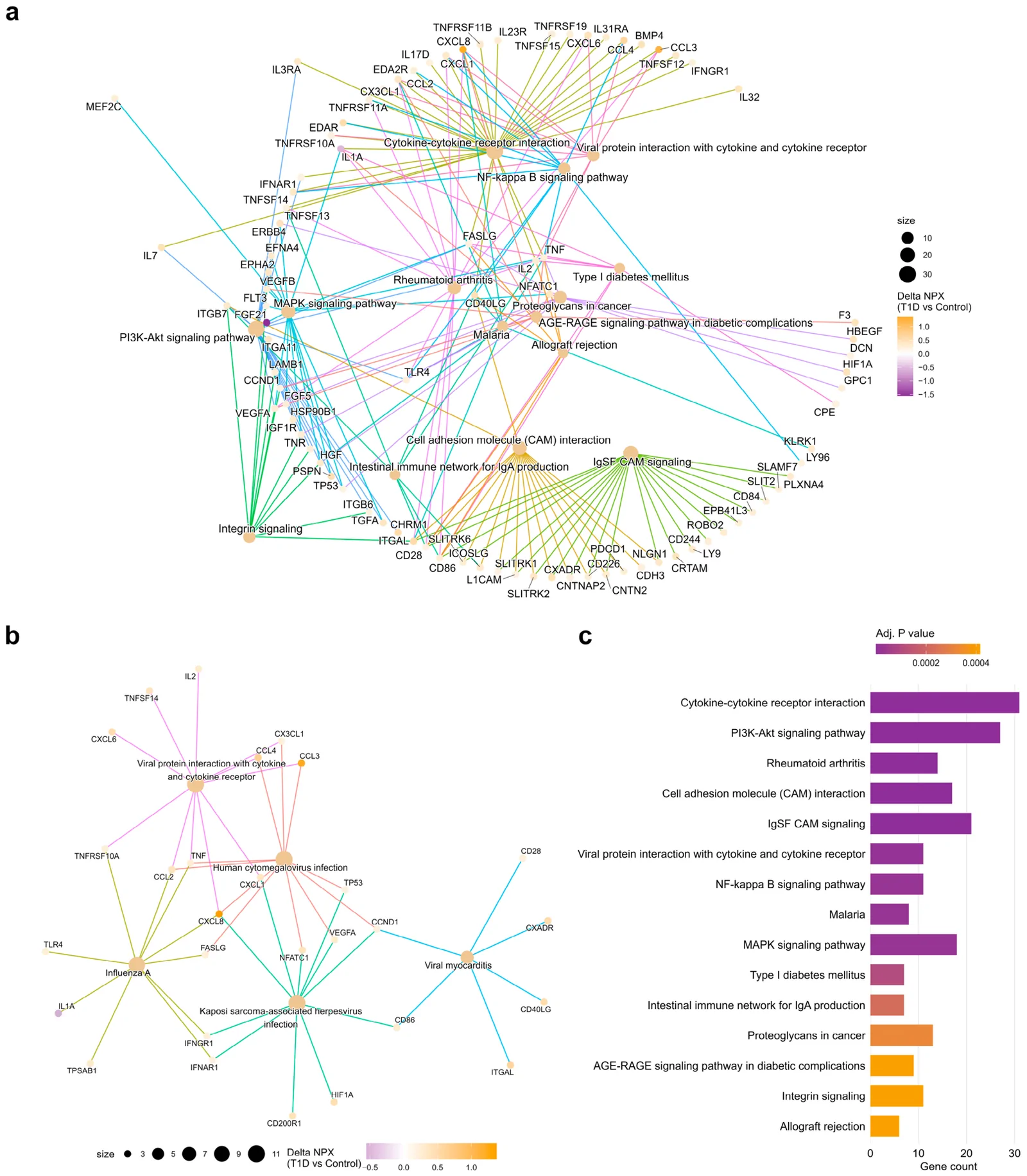
Maternal serum immune networks and infection-annotated pathways at delivery. (**a**) Pathway–gene network of maternal serum proteins at delivery showing the top enriched KEGG pathways in mothers of children who later developed T1D compared with control mothers. Nodes represent proteins and pathways; edges indicate membership of proteins in specific pathways. Enriched pathways include cytokine–cytokine receptor interaction, viral protein interaction with cytokine and cytokine receptor, NF-κB signaling, MAPK signaling, PI3K–Akt signaling, cell adhesion molecule (CAM) interaction, and integrin signaling. (**b**) Focused maternal pathway–gene network restricted to KEGG pathways annotated as infection or host–pathogen interaction. Enriched pathways include viral protein interaction with cytokine and cytokine receptor, viral myocarditis, human cytomegalovirus infection, influenza A, and Kaposi sarcoma-associated herpesvirus infection. As in the neonatal network, virus-associated pathways converge on shared inflammatory mediators rather than forming isolated pathogen-specific signatures. (**c**) Enrichment of the 15 KEGG pathways shown in panel a, ranked by adjusted *p* value; bar length shows the number of proteins in each pathway. Enrichment was based on nominally significant proteins (*p* < 0.05).

Maternal serum proteomics at delivery, which showed a stronger statistical signal than neonatal serum (60 proteins at FDR < 0.05), revealed a partially overlapping immune profile in mothers of children who later developed T1D (Supplementary Figures S1–S4). KEGG pathway enrichment analysis showed enrichment of inflammatory, cytokine-mediated, and infection-related immune pathways, including cytokine–cytokine receptor interaction, viral protein interaction with cytokine and cytokine receptor, NF-κB signaling, MAPK signaling, PI3K–Akt signaling, and type I diabetes mellitus. Additional enriched pathways included rheumatoid arthritis and the AGE-RAGE signaling pathway in diabetic complications, reflecting broader autoimmune and metabolic disease associations within the maternal proteomic signature (Figure 2a).

Network analysis showed that these pathways converged on interconnected signaling modules centered on cytokine-mediated signaling together with NF-κB, MAPK, and PI3K–Akt pathways. Cell adhesion- and CAM-related pathways were also prominently represented, linking inflammatory signaling with immune communication pathways and leukocyte trafficking (Figure 2a,c). In a permutation test, a STRING protein–protein interaction network built from the maternal differentially abundant proteins was significantly more connected than networks built from 5000 random protein sets (*p* = 0.0028; Supplementary File S2).

To further examine infection-related immune signatures, the KEGG enrichment results were restricted to pathways associated with infection and host–pathogen interactions. This analysis identified a focused subnetwork comprising viral protein interaction with cytokine and cytokine receptor, viral myocarditis, human cytomegalovirus infection, influenza A, and Kaposi sarcoma-associated herpesvirus infection (Figure 2b). Similar to the neonatal network, these virus-associated pathways converged on a shared set of inflammatory and immune signaling molecules (Figure 2b).

### 3.3. Shared Immune Hubs Link Maternal and Neonatal Immune Networks at Birth

**Figure 3:**
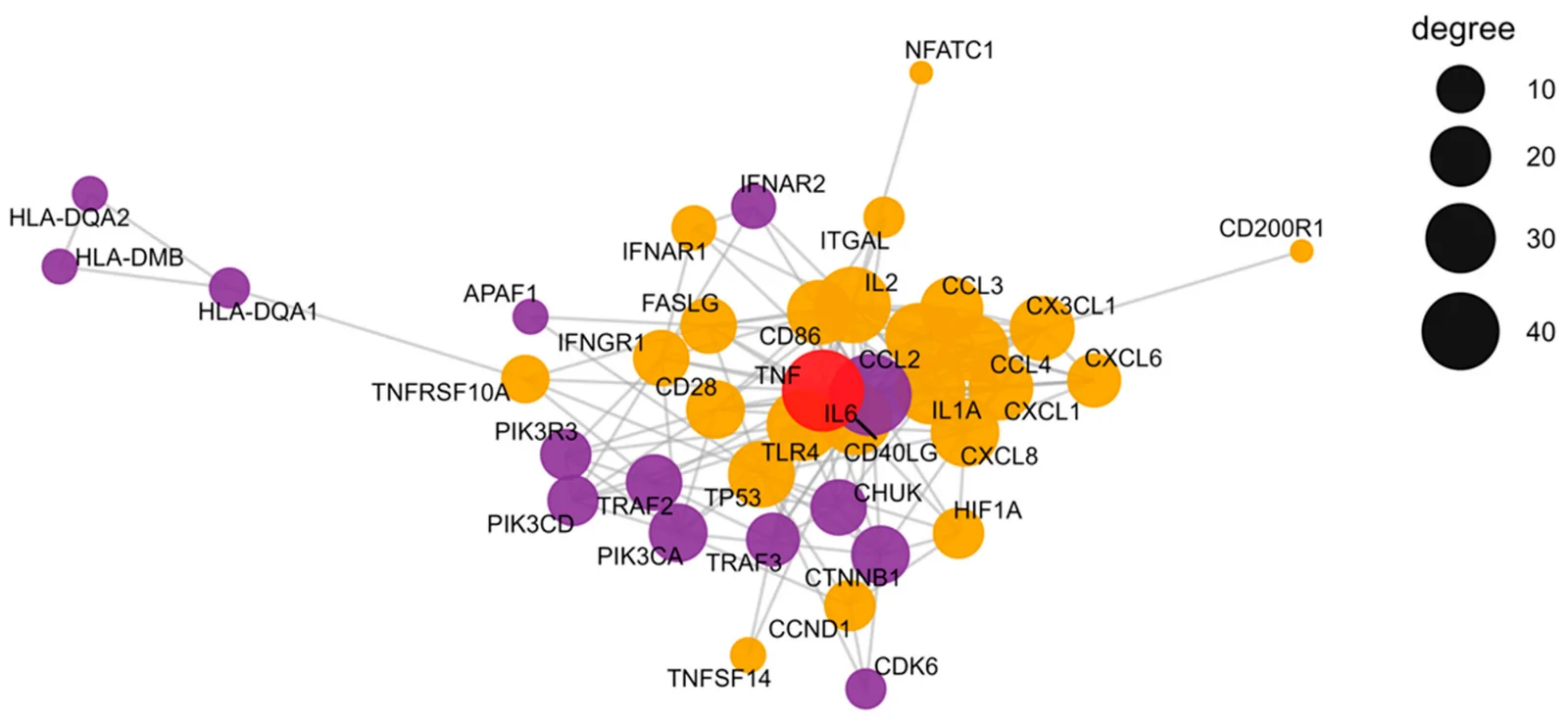
Integrated maternal and neonatal immune-related protein interaction network at birth. Protein–protein association network generated using STRING by integrating the proteins identified in the maternal and neonatal proteomic analyses. Nodes are colored by origin: maternal (orange), neonatal (purple), or shared (red), and node size reflects degree centrality. The network highlights an interconnected inflammatory core involving TNF, IL6, CD40LG, TLR4, CCL/CXCL chemokines, and interferon-related signaling components. Because maternal and neonatal samples were derived from independent cohort groups rather than matched mother–child dyads, the network reflects shared pathway involvement at the population level and does not indicate relationships between mothers and their own children.

To examine the relationship between maternal and neonatal immune signatures, we constructed an integrated protein interaction network by combining proteins identified in the maternal (orange) and neonatal (purple) infection- and inflammation-associated networks (Figure 3). TNF was identified in both datasets (red) and occupied a central position within the network. The resulting network revealed an interconnected immune module centered on shared inflammatory mediators, including TNF, IL6, CD40LG, TLR4, CCL2, CCL3, CCL4, CXCL1, CXCL6, and CXCL8, together with interferon-related signaling components. This module linked the maternal and neonatal subnetworks.

Although several proteins were unique to either the maternal or the neonatal datasets, they remained connected through this common inflammatory core rather than forming completely separate subnetworks. In contrast, HLA-associated proteins were primarily confined to the neonatal component, forming a more distinct cluster while maintaining indirect connections to the central inflammatory network (Figure 3).

### 3.4. Integrative Analysis Highlights Shared Maternal and Neonatal Immune Modules Centered on Inflammatory and Infection-Related Pathways

**Figure 4:**
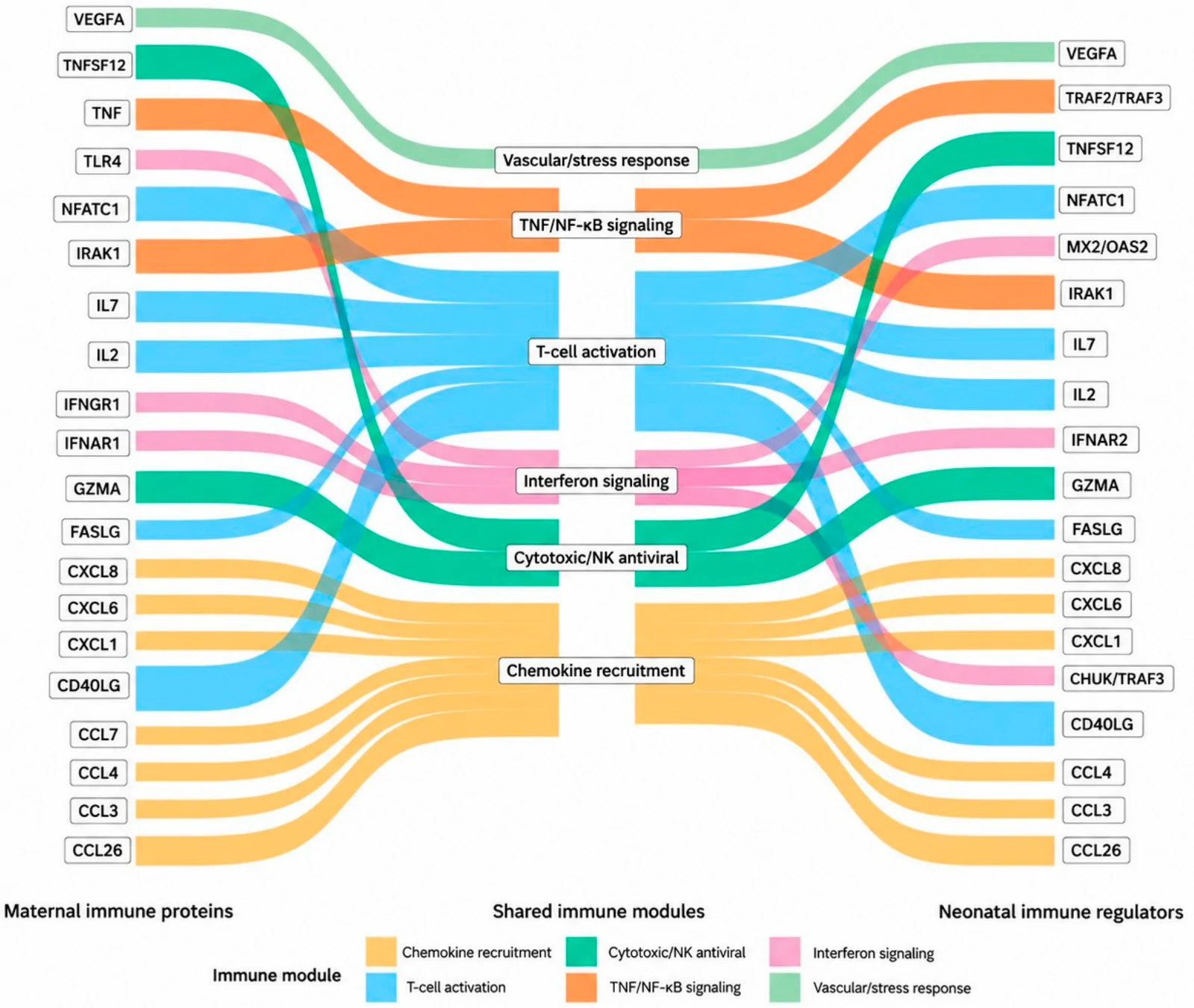
Shared maternal and neonatal immune signatures at birth in future T1D. Sankey diagram illustrating convergence between maternal immune proteins (left), shared immune modules (center), and neonatal immune regulators (right). Shared immune modules include chemokine-mediated recruitment, T-cell activation, interferon signaling, TNF/NF-κB signaling, cytotoxic/NK-cell response, and vascular/stress response. Flows illustrate how maternal proteins map to shared functional modules and how these modules connect to neonatal immune regulators, highlighting convergent immune-related patterns across maternal and neonatal datasets from independent cohort groups.

To summarize cross-compartment convergence, we integrated maternal immune proteins, shared immune modules, and neonatal immune regulators into a Sankey-style diagram. Six functional modules were defined: chemokine-mediated recruitment, T-cell activation, cytotoxic/NK-cell response, interferon signaling, TNF/NF-κB signaling, and vascular/stress response (Figure 4).

Multiple maternal proteins converged on these common immune modules, which were also linked to corresponding neonatal proteins and genes. Chemokine-mediated recruitment represented the largest shared module, connecting multiple members of the CCL and CXCL families across both maternal and neonatal datasets. Similarly, the TNF/NF-κB and interferon signaling modules integrated inflammatory mediators from both compartments. The T-cell activation and cytotoxic/NK-cell response modules further linked adaptive and innate immune pathways into a common network (Figure 4).

### 3.5. Epigenetic Alterations Map to Shared Immune and Infection-Related Pathways

**Figure 5:**
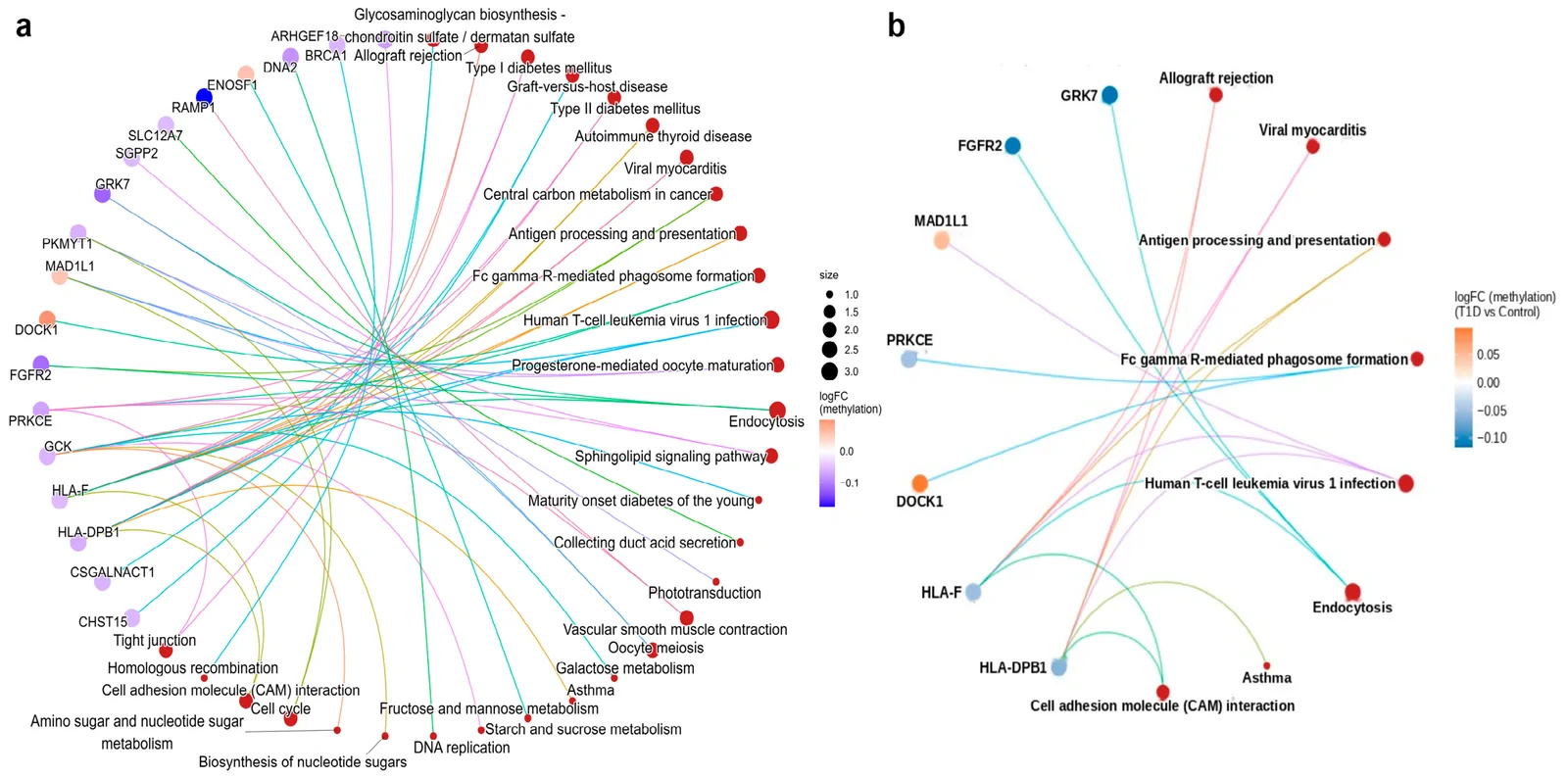
Cord blood DNA methylation and immune/infection-related pathways. (**a**) Pathway–gene network of differentially methylated genes (DMGs) in neonatal cord blood comparing future T1D cases and controls (*p* ≤ 0.05). Nodes represent genes and pathways; edges indicate membership of genes in specific pathways. Enriched pathways include antigen processing and presentation, Fc gamma R-mediated phagosome formation, cell adhesion molecule interaction, viral myocarditis, and human T-cell leukemia virus 1 infection. (**b**) Focused DMG pathway–gene network restricted to immune- and infection-associated KEGG pathways. Multiple virus-related pathways converge on a limited set of shared DMGs, including HLA-DPB1, HLA-F, DOCK1, PRKCE, MAD1L1, FGFR2, and GRK7, which connect several immune and host–pathogen pathways.

In contrast to the broad proteomic signatures, analysis of cord blood DNA methylation identified only a limited number of DMGs between future T1D cases and control subjects. Nevertheless, pathway enrichment analysis of these DMGs identified immune- and infection-related KEGG pathways, including antigen processing and presentation, phagosome formation, cell adhesion molecule (CAM) interaction, viral myocarditis, and human T-cell leukemia virus 1 infection (Figure 5a).

A focused pathway–gene network restricted to immune- and infection-associated pathways further showed that multiple virus-associated pathways converged on a limited set of shared DMGs rather than representing distinct pathogen-specific signatures (Figure 5b). Several DMGs, including HLA-DPB1, HLA-F, DOCK1, PRKCE, MAD1L1, FGFR2, and GRK7, connected multiple pathways, highlighting common immune regulatory processes underlying these enrichments.

## 4. Discussion

In this exploratory study, we identified evidence of related maternal and neonatal immune signatures at birth associated with later T1D. Both proteomic analyses highlighted inflammatory and infection-related immune processes involving cytokine signaling, NF-κB activation, chemokine-mediated recruitment, T-cell responses, and interferon-related pathways. The maternal findings were statistically stronger, with 60 proteins remaining significant after FDR correction and a protein interaction network more connected than expected by chance, whereas the neonatal findings were based mainly on nominally significant proteins, and the neonatal network was not significantly more connected than random networks; the neonatal findings should therefore be regarded as hypothesis-generating. Network integration identified shared hub proteins, including TNF, CD40LG, TLR4, and multiple CCL and CXCL chemokines. These proteins orchestrate innate and adaptive immune responses and play important roles in both antiviral defense and autoimmune inflammation. Cord blood DNA methylation provided complementary, although less pronounced, evidence pointing toward similar immune and host–pathogen-related processes.

The contribution of viral infections to the development of T1D has been investigated for decades. Nearly a century ago, Gundersen first suggested a possible link between mumps infection and diabetes onset [28]. Subsequent work by Yoon et al. provided direct evidence for virus-induced diabetes by isolating a diabetogenic virus from the pancreas of a child with diabetic ketoacidosis [13]. However, despite substantial epidemiological and experimental evidence linking viral infections to T1D, no single virus has been consistently identified as the causal trigger of the disease [10,11]. Enteroviruses have been strongly implicated in T1D in previous epidemiological and mechanistic studies [29]. In the present study, however, enterovirus-related pathways were not identified among the enriched pathways. A recent systematic review and meta-analysis of controlled observational studies found significant associations between enterovirus detection and both islet autoimmunity and T1D, while also highlighting substantial heterogeneity across studies [30]. In the prospective TEDDY cohort, prolonged Enterovirus B shedding was associated with the development of islet autoimmunity, although this association was not observed for T1D itself [31]. Thus, increasing evidence suggests that different viral infections may contribute to T1D development rather than a single specific virus accounting for disease risk, particularly in genetically susceptible individuals [10,11].

Our findings are consistent with this broader concept. The maternal and neonatal serum proteomic profiles showed enrichment of multiple virus-associated KEGG pathways, including viral myocarditis, cytomegalovirus, Epstein–Barr virus, hepatitis B, herpes simplex virus 1, coronavirus, and influenza.

In addition, maternal and neonatal serum proteomic profiles shared several inflammatory and antiviral immune processes, including type I interferon signaling, NF-κB/TNF activation, and chemokine-mediated leukocyte recruitment. These pathways are canonical components of antiviral immunity and are also implicated in β-cell-directed autoimmunity [21]. Mechanistic studies show that viral recognition by pattern-recognition receptors activates NF-κB and interferon-regulatory factors, triggering the production of type I interferons and chemokines that recruit immune cells to the pancreatic islets [21]. The presence of these pathways in our maternal and neonatal proteomic datasets suggests that antiviral immune signatures may be detectable at birth in relation to later T1D. However, these findings reflect shared immune signaling pathways and do not establish the presence of specific viral infections or a direct mechanistic link to subsequent islet autoimmunity.

The simultaneous involvement of T-cell activation and cytotoxic/NK-cell modules further suggests that these immune signatures encompass both adaptive and innate immune processes. Together, these patterns may reflect an antiviral-like inflammatory state or a heightened state of immune readiness early in life.

In contrast to the broad proteomic findings, cord blood DNA methylation changes in the overall case–control comparison were relatively modest. Only a limited number of nominally significant differentially methylated genes (DMGs; *p* ≤ 0.05) were identified. Nevertheless, pathway enrichment analysis of these DMGs identified inflammatory and immune-related pathways, including pathways annotated as virus-associated, consistent with patterns observed in the maternal and neonatal proteomic datasets. In particular, pathway network analysis identified several shared DMGs, including HLA-DPB1, HLA-F, DOCK1, PRKCE, MAD1L1, FGFR2, and GRK7, which linked multiple immune and host–pathogen interaction pathways. This suggests convergence on common immune regulatory mechanisms. Although DNA methylation and serum protein levels were both measured at birth, they reflect distinct biological processes and timescales. Prenatal DNA methylation patterns may influence the regulation of immune-related genes, while circulating protein levels reflect immune activity at the time of sampling.

Our results suggest that related maternal and neonatal immune signatures may represent an early biological framework through which prenatal exposures influence later T1D risk. They may also guide future studies of the potential contribution of antiviral immune responses to T1D development. Rather than indicating exposure to a single specific pathogen, the observed patterns may reflect a broader prenatal immune profile, detectable at birth, associated with increased susceptibility to T1D and potentially modified by genetic background. This is consistent with epidemiological studies linking maternal infection during pregnancy to increased offspring T1D risk [17], as well as with evidence linking early childhood respiratory tract infections to T1D risk [32].

The related maternal and neonatal immune signatures observed in the present study may represent the immunological consequences of maternal immune activation, inherited susceptibility to inflammatory dysregulation, or a combination of these factors. Because our analyses did not directly measure maternal infection, maternal symptoms, or fetal viral infection, these potential mechanisms cannot be distinguished.

This study has several limitations. The sample sizes were modest relative to the number of protein assays evaluated, limiting statistical power, particularly for detecting small differences in protein abundance (a sensitivity analysis of statistical power is provided in Supplementary File S1, Table S1). Although multiple-testing correction was applied to the proteomic analyses, many findings did not remain significant after false discovery rate (FDR) correction, particularly in the neonatal dataset. In addition, the overall cord blood DNA methylation signal was modest.

Although this study integrated maternal serum proteomic, neonatal serum proteomic, and cord blood DNA methylation data within a prospective birth cohort, the maternal and neonatal proteomic analyses were conducted in independent groups rather than in matched mother–child pairs. Therefore, the overlap in maternal and neonatal protein signatures, including the network in Figure 3, cannot establish direct maternal–offspring relationships. Furthermore, HLA genotype data were not consistently available across study groups and could not be included as a covariate in all analyses.

An additional limitation is that maternal and neonatal serum proteomes were measured using different Olink platforms. Although each dataset was analyzed separately, differences in assay chemistry, protein coverage, and normalization procedures preclude direct quantitative comparisons of NPX values across platforms. Accordingly, the integrated STRING network should be interpreted as a representation of known or predicted protein associations rather than as quantitative relationships between the datasets. Direct measures of maternal or fetal viral infection were also not available, precluding conclusions regarding virus-specific prenatal programming.

The cord blood DNA methylation gene list was derived from a previous publication by our group and was not re-derived in the present study. Differences in analytical procedures may limit direct comparability between the methylation and proteomic findings. Therefore, their integration should be viewed as a qualitative assessment of biological convergence.

Finally, the proteomic findings lack independent validation. Although the high-throughput proteomic analyses identified immune-related patterns associated with future T1D, these findings require replication in larger, independent cohorts and targeted experimental validation of selected proteins, for example, using Western blot or other appropriate orthogonal methods. Such validation would help assess the robustness of the observed protein-level differences and clarify their potential relevance to T1D development.

Future studies designed to address these limitations should include paired maternal–neonatal samples and, where feasible, placental tissue and fetal material, together with more complete HLA genotype information and direct measures of maternal or fetal infection. Such studies could further clarify maternal–fetal immune interactions and help distinguish generalized prenatal inflammatory processes from virus-related immune responses.

In addition, longitudinal and multigenerational studies incorporating epigenetic analyses could investigate whether prenatal environmental exposures are associated with persistent epigenetic changes and potential health effects across subsequent generations.

## 5. Conclusions

Our findings suggest that immune-related protein profiles differ at birth in both maternal and neonatal serum in relation to later T1D in the child. The maternal findings were statistically more robust, with 60 proteins remaining significant after FDR correction, and the connectivity of the protein–protein interaction network exceeding random expectation. The neonatal findings were based mainly on nominally significant proteins, and the connectivity of the neonatal network did not significantly exceed random expectation; these findings should therefore be regarded as hypothesis-generating. These profiles involve inflammatory and immune-related pathways that may be associated with the maternal immune environment. By integrating maternal and neonatal serum proteomics with cord blood DNA methylation findings, this study highlights overlapping immune-related features across maternal and neonatal profiles. These findings may help inform the design of future studies aimed at clarifying the biological significance of these profiles and their potential relevance to early risk assessment and prevention.

## Data Availability

The data presented in this study are available on request from the corresponding author.

## Supplementary Materials

The following supporting information can be downloaded at: https://www.mdpi.com/article/10.3390/cells15212036/s1. File S1: Supplementary materials and methods, including Table S1 and Figures S1–S4; Figure S1: PCA of neonatal serum proteomic profiles; Figure S2: PCA of maternal serum proteomic profiles at delivery; Figure S3: Volcano plot of differentially abundant proteins between mothers of children who later developed T1D and control mothers; Figure S4: Boxplots of the top differentially abundant maternal serum proteins; Table S1: Sensitivity analysis of statistical power for the maternal and neonatal serum proteomic comparisons; Table S2: Differentially methylated genes (DMGs) imported from the previously published cord blood epigenome-wide analysis of the ABIS cohort; Table S3: Differentially abundant proteins in neonatal and maternal serum; Table S4: Proportions of missing values for individual proteins in the maternal and neonatal serum proteomic datasets; Table S5: Assignment of proteins to the six immune modules in Figure 4, with the corresponding KEGG pathways; File S2: Network permutation analysis of the maternal and neonatal protein interaction networks compared with random protein sets. References [22,23,33,34,35,36,37,38,39,40,41,42,43] are cited in the Supplementary Materials.
