# Supplementary material for: Maternal and Neonatal Immune Signatures at Birth and Future Type 1 Diabetes: Potential Antiviral and Inflammatory Immune Profiles
Source: Cells. 2026 Nov 9;15(21):2036. doi: 10.3390/cells15212036 (PMC13761635; doi:10.3390/cells15212036)
Supplement: Supplementary file 1 [file cells-15-02036-s001.zip › Supplementary File S1/cells-4623614-Table S1 and Figure S1-S4.pdf]

Maternal and Neonatal Immune signatures at Birth in Future Type 1 Diabetes: Potential Antiviral and Inflammatory Immune Signatures

Shamila D. Alipoor [1], Sheida Ardeshirilajimi [1] and Johnny Ludvigsson [1,2]

[1] Division of Pediatrics, Department of Biomedical and Clinical Sciences, Linköping University, Linköping, Sweden
[2] Crown Princess Victoria Children's Hospital, Linköping University Hospital, Linköping, Sweden
* Correspondence: **to:** Johnny Ludvigsson, Division of Pediatrics, Department of Biomedical and Clinical Sciences, Linköping University, Linköping, Sweden (email: Johnny.Ludvigsson@liu.se)

Supplementary material and methods:

Cohort characteristics

ABIS cohort [1, 2] follows, to present day, 17,055 individuals born between October 1997 and 1999 in Southeastern Sweden. Families were recruited from nine obstetric clinics across all hospitals in the counties of Östergötland, Småland, Blekinge, and Öland. They received oral and written information, and were also offered video-information, and were then asked to join the study. Out of the 21,700 children born in the region, 78.6% of families provided informed consent for their child to participate. Parents completed extensive questionnaires and diaries from the child's birth through >20 years of age, with ABIS children themselves answering questionnaires at later visits. Cord blood samples were collected from all ABIS children. The ABIS register is connected to the Swedish National Patient Register giving diagnosis of diseases, and to the National Drug Prescription Register which has been used for validation of diagnosis of T1D.

Ethical Statement

Ethical approval for ABIS was obtained by Research Ethics Committees of Faculty of Health Science at Linköping Univ., Ref. 1997/96287 and 2003/03-092 and the Medical

Faculty of Lund University Dnr 99227, Dnr 99321. Connection to National Registers was approved (Dnr 03-513 and 2018- 318-32).

DNA extraction

Genomic DNA was isolated from whole blood using the QIAamp DNA Mini Kit on the QIAcube Connect automated platform (Qiagen, Germany). DNA concentration was measured with the Quantus Fluorometer and QuantiFluor ONE dsDNA System (E4871, Promega, USA), and DNA quality was evaluated using a NanoDrop ND-1000 spectrophotometer (Thermo Fisher Scientific, USA).

Methylation array data

Genomic DNA (250 ng/sample) samples were bisulfite-converted using EZ DNA Methylation Kit (Zymo Research) and profiled using the Illumina MethylationEPIC v1.0 array. BeadChips were scanned on an Illumina NextSeq 550.

Data preprocessing and quality control

Methylation signal intensities were extracted from raw IDAT files using the minfi R package (v1.50.0) in R version 4.4.3[3] [4]. During the initial preprocessing stage, signal intensities were normalized and converted to β-values using the intra-sample Beta-Mixture Quantile (BMIQ) normalization[5] procedureand the inter-array Gaussian Mixture Quantile Normalization (GMQN) method[6] implemented in the GMQN R package (v0.1.0) [6].

Probe-level quality control and filtering were applied in the subsequent steps. Probes with missing values in more than 25% of the samples were excluded. Additionally, cross reactive probes, non-CpG probes, probes mapping to known single nucleotide polymorphisms (SNPs), and probes located on sex chromosomes (X and Y) were discarded using the ChAMP R package (v2.34.0) [4] . Remaining missing values were

imputed using k-nearest neighbor (kNN) imputation via the scikit-learn Python package[7]

To identify and correct technical artifacts, singular value decomposition (SVD) analysis was performed using a modified version of the SVD function from the ChAMP R package. Major sources of variation significantly associated ($P < 0.05$) with SVD components explaining

$> 3\%$ of the variance, such as slide and array effects, were adjusted using the ComBat algorithm from the sva R package (v3.52.0) [8]. A follow-up SVD analysis confirmed successful artifact removal.

Finally, data quality was comprehensively evaluated using density plots of β-value distributions. Principal component analysis (PCA) was performed to visualize sample clustering and confirm preprocessing efficiency.

Differential DNA methylation analysis

Differentially methylated CpGs (DMCs) between groups were identified using the *limma R* package (v3.60.6) [9]. For each CpG, linear models were fitted adjusting for sex and cell type proportions that differed significantly between groups ($P < 0.05$). Empirical Bayes moderation was applied to stabilize variance estimation, and differential methylation was assessed using moderated t-statistics. We applied a P value cutoff of 0.05 and a $|\text{logFC}|$ cutoff of 0.05 to determine whether a CpG was differentially methylated [10, 11]. The identified DMCs were mapped to corresponding differentially methylated genes (DMGs) using Illumina EPIC array annotations *ilm10b4.hg19*[12]. The results were visualized as heatmaps using *ComplexHeatmap* package (v2.10.0)[10, 11] and as volcano plots using *ggplot2*[13] in R(v3.4.4).

Statistics:

Differential DNA methylation was evaluated with limma using moderated t-statistics, and CpG sites were considered differentially methylated at $P \le 0.05$ and $|\text{logFC}| \ge$

0.05. For pathway enrichment, significance was set at $P < 0.05$. For Olink data, group differences were evaluated using limma with empirical Bayes moderation. Proteins with nominal $p < 0.05$ and $|\Delta NPX| \geq 0.2$ were considered differentially abundant, and Benjamini–Hochberg FDR-adjusted p values are reported (supplementary table 3).

Sensitivity Analysis of Statistical Power

A sensitivity analysis was performed for the maternal and neonatal datasets to evaluate statistical power across different effect sizes (Cohen's d = 0.2, 0.3, 0.5, and 0.8) and significance levels (α = 0.05, 0.01, and 0.001). The minimum detectable effect size for 80% power was also estimated at α = 0.05.

Table S1. Sensitivity analysis of statistical power for maternal and neonatal serum proteomic comparison

Dataset	Cases / controls	d = 0.2	d = 0.3	d = 0.5	d = 0.8	Minimum detectable Cohen's d (80% power)
Maternal, α = 0.05	24 / 38	0.12	0.20	0.47	0.86	0.74
Maternal, α = 0.01	24 / 38	0.03	0.07	0.24	0.66	0.92
Maternal, α = 0.001	24 / 38	0.01	0.01	0.07	0.36	1.13
Neonatal, α = 0.05	32 / 48	0.14	0.25	0.58	0.93	0.65
Neonatal, α = 0.01	32 / 48	0.04	0.10	0.33	0.80	0.80

Dataset	Cases / controls	d = 0.2	d = 0.3	d = 0.5	d = 0.8	Minimum detectable Cohen's d (80% power)
Neonatal, $\alpha = 0.001$	32 / 48	0.01	0.02	0.12	0.54	0.98

Figure S1. PCA of neonatal proteomic data

Principal component analysis (PCA) was performed to assess the overall structure of neonatal cord-blood proteomic profiles in the T1D and control groups. Samples are colored according to disease group. PC1 and PC2 explained 19.4% and 11.5% of the total variance,

respectively. No samples were excluded based on their position in the PCA plots.

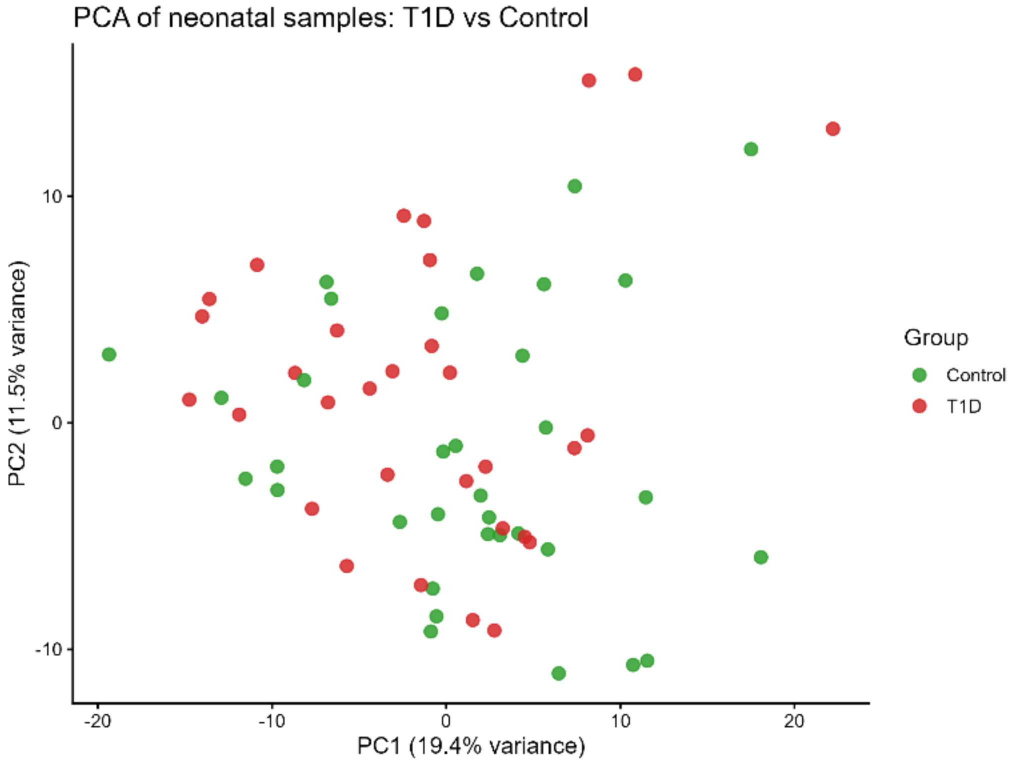

Figure S2. PCA of maternal birth serum proteomic profiles

PCA of Olink NPX data from 62 mothers after quality-control procedures, including 24 mothers whose children subsequently developed type 1 diabetes (T1D) and 38 control mothers. Samples are colored according to group. PC1 and PC2 explain 20.3% and 9.9% of the total variance, respectively. No sample exceeded the outlier threshold (PC1 or PC2 score > 3

SD from the mean). One sample (18663; PC1 = −3.02 SD) marginally exceeded this threshold; it passed all Olink QC criteria and was retained in the primary analysis. In a sensitivity analysis excluding this sample, 46 of the 60 FDR-significant proteins remained significant at FDR < 0.05 (the remainder at FDR < 0.08), all with concordant direction of effect and highly similar effect sizes (r = 0.998), and the top-ranked proteins were unchanged.

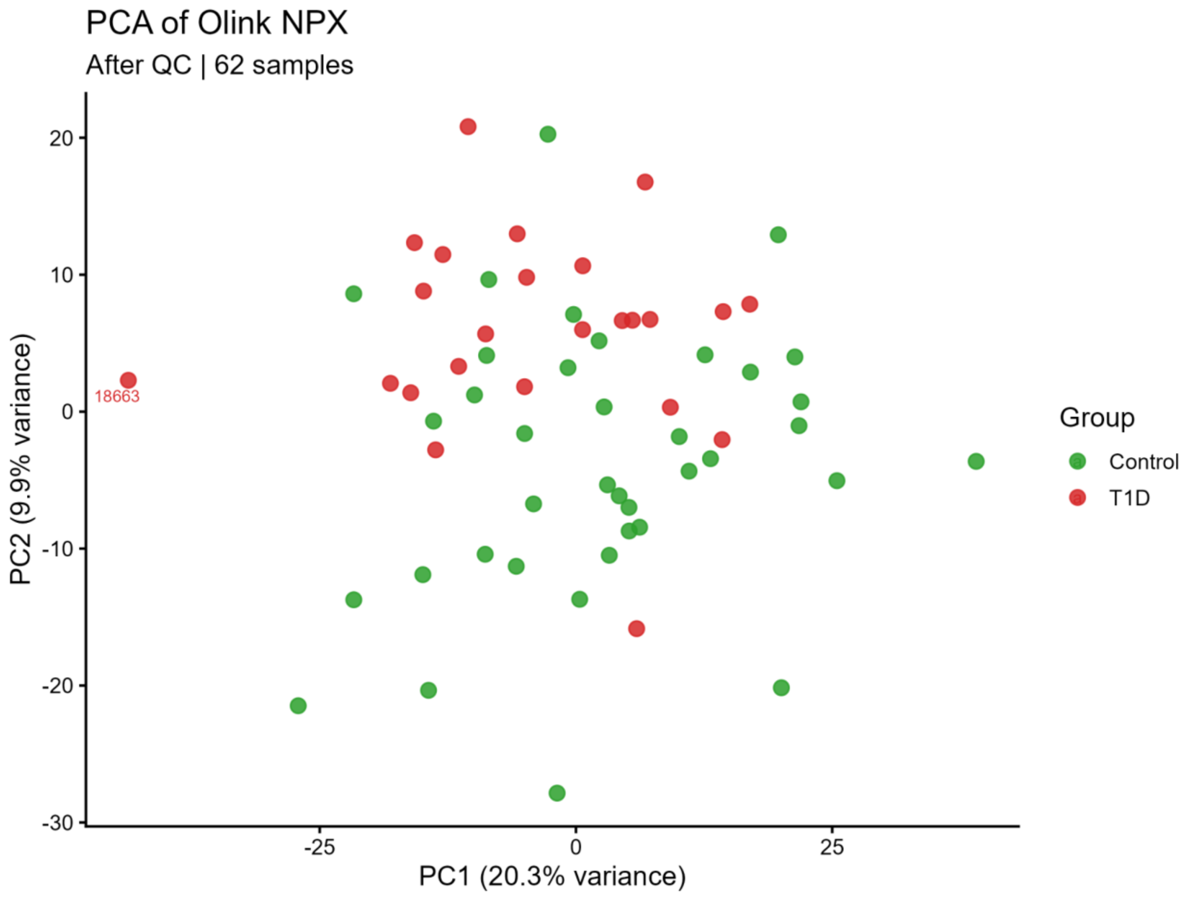

Figure S3: Volcano plot of differentially abundant proteins between mothers of children who later developed type 1 diabetes (T1D) and control mothers. Proteins are plotted according to ΔNPX (log2 fold change) and −log10(FDR).

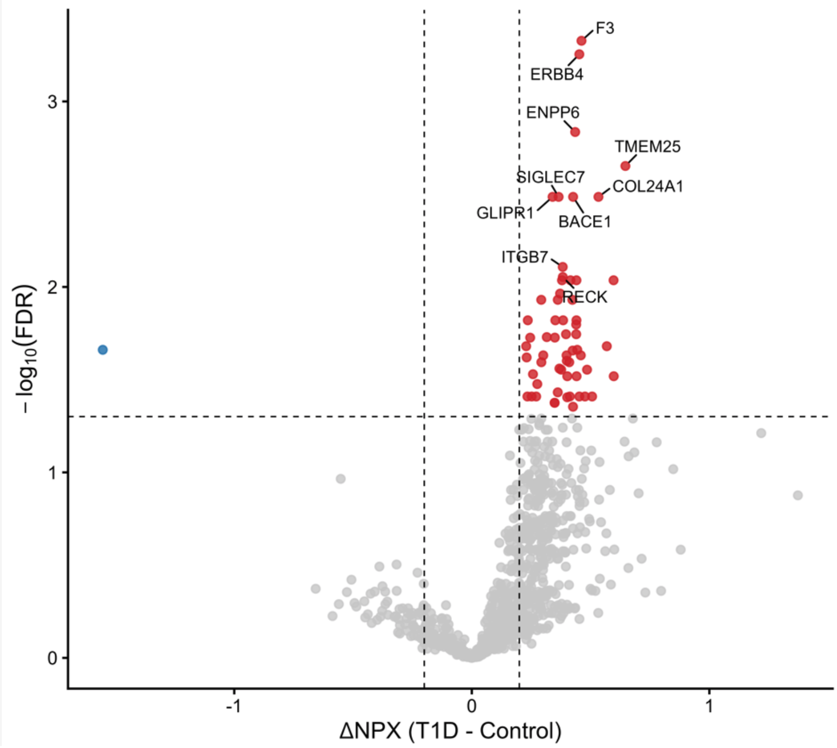

Figure S4: Boxplots of the top differentially abundant proteins identified by Olink proteomics. NPX values are shown for mothers of children who later developed T1D and control mothers.

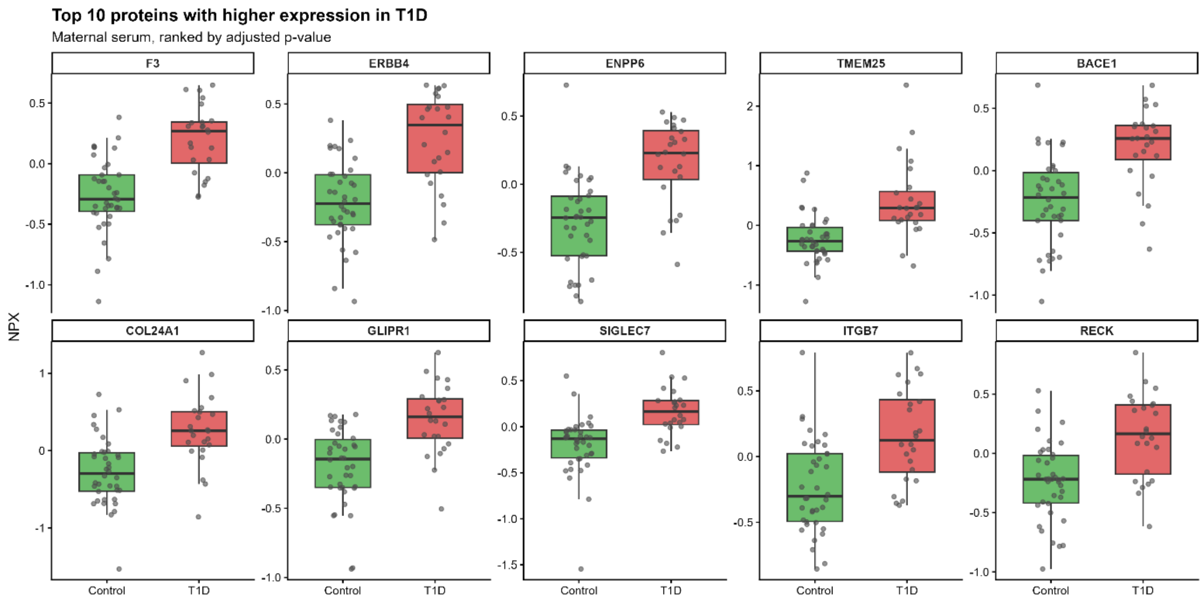

References:

1. Ludvigsson J, Ludvigsson M, Sepa A (2001) Screening for prediabetes in the general child population: maternal attitude to participation. Pediatric Diabetes 2(4): 170-174
2. Nygren M, Carstensen J, Koch F, Ludvigsson J, Frostell A (2015) Experience of a serious life event increases the risk for childhood type 1 diabetes: the ABIS population-based prospective cohort study. Diabetologia 58: 1188-1197
3. Team R (2017) R: A language and environment for statistical com-puting. R Foundation for Statistical Computing, Vienna, Austria URL https://www R-project org
4. Tian Y, Morris TJ, Webster AP, et al. (2017) ChAMP: updated methylation analysis pipeline for Illumina BeadChips. Bioinformatics 33(24): 3982-3984
5. Teschendorff AE, Marabita F, Lechner M, et al. (2013) A beta-mixture quantile normalization method for correcting probe design bias in Illumina Infinium 450 k DNA methylation data. Bioinformatics 29(2): 189-196
6. Xiong Z, Li M, Ma Y, Li R, Bao Y (2022) GMQN: a reference-based method for correcting batch effects and probe bias in HumanMethylation BeadChip. Frontiers in genetics 12: 810985
7. Pedregosa F, Varoquaux G, Gramfort A, et al. (2011) Scikit-learn: Machine learning in Python. the Journal of machine Learning research 12: 2825-2830
8. Johnson WE, Li C, Rabinovic A (2007) Adjusting batch effects in microarray expression data using empirical Bayes methods. Biostatistics 8(1): 118-127
9. Ritchie ME, Phipson B, Wu D, et al. (2015) limma powers differential expression analyses for RNA-sequencing and microarray studies. Nucleic acids research 43(7): e47-e47
10. Gu Z (2022) Complex heatmap visualization. Imeta 1(3): e43
11. Gu Z, Eils R, Schlesner M (2016) Complex heatmaps reveal patterns and correlations in multidimensional genomic data. Bioinformatics 32(18): 2847-2849

12. Hansen K (2017) IlluminaHumanMethylationEPICanno. ilm10b4. hg19: Annotation for Illumina's EPIC methylation arrays. R package version 06 0
13. Wickham H, Chang W, Wickham MH (2016) Package 'ggplot2'. Create elegant data visualisations using the grammar of graphics Version 2(1): 1-189
