# Supplementary material for: Maternal and Neonatal Immune Signatures at Birth and Future Type 1 Diabetes: Potential Antiviral and Inflammatory Immune Profiles
Source: Cells. 2026 Nov 9;15(21):2036. doi: 10.3390/cells15212036 (PMC13761635; doi:10.3390/cells15212036)
Supplement: Supplementary file 1 [file cells-15-02036-s001.zip › Supplementary File S2/cells-4623614-File S2.pdf]

Network Permutation Analysis

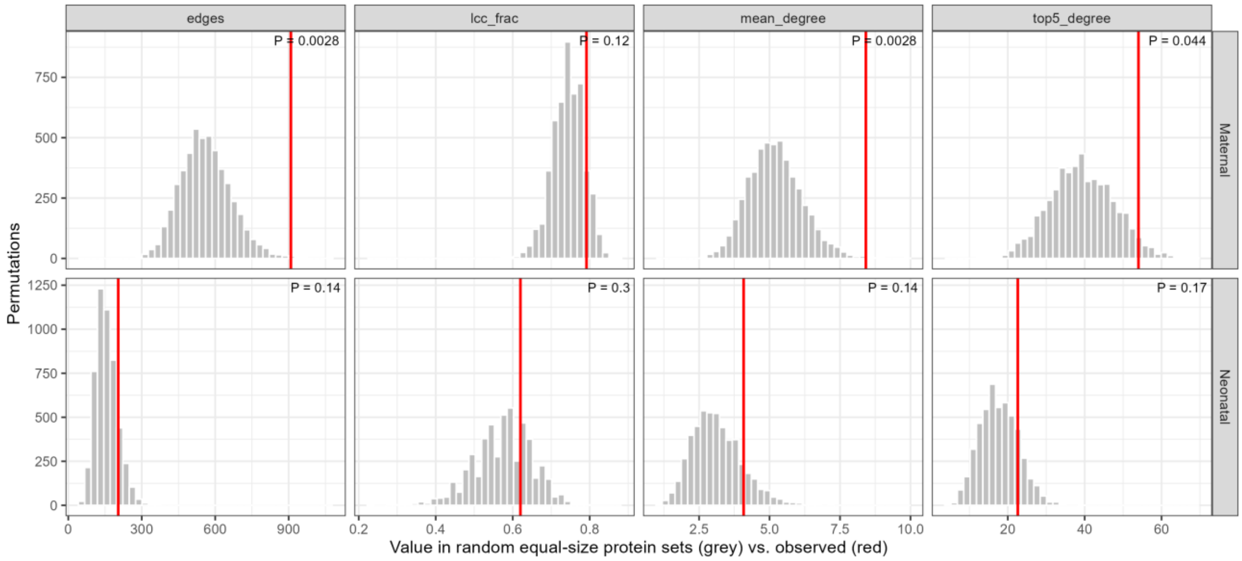

Network connectivity of the observed protein sets compared with random protein sets. Distribution of four network statistics (edges; fraction of proteins in the largest connected component, lcc_frac; mean degree; and top-five hub degree, top5_degree) in 5,000 STRING networks built from random, equally sized protein sets drawn from the proteins analysed on the corresponding Olink platform (grey histograms). The red line shows the value in the observed network. Upper row, maternal serum; lower row, neonatal serum . Empirical P-values are shown in each panel (P = (r + 1)/(n + 1)). The maternal network was more connected than expected by chance, whereas the neonatal network did not differ from random sets. These analyses are exploratory.

Table Sa: Network statistics of the observed protein sets versus random protein sets. Observed and null values of six network statistics for the maternal and neonatal networks (5,000 random sets of equal size). Null 95th pct, 95th percentile of the null distribution. Empirical P = (r + 1)/(n + 1). Values with P < 0.05 are in bold.

Network	Statistic	Observed	Null mean	Null 95th pct	Empirical P
Maternal	Edges	909	562.95	739.05	**0.0028**
Maternal	Density	0.0391	0.0242	0.0318	**0.0028**
Maternal	Mean degree	8.42	5.21	6.84	**0.0028**
Maternal	Max degree	77	54.70	73	**0.021**
Maternal	Top-5 hub degree	54	39.40	53.4	**0.044**
Maternal	Largest component (fraction)	0.792	0.744	0.806	0.123
Neonatal	Edges	204	156.61	237	0.140
Neonatal	Density	0.0412	0.0316	0.0479	0.140
Neonatal	Mean degree	4.08	3.13	4.74	0.140
Neonatal	Max degree	25	25.26	37	0.551
Neonatal	Top-5 hub degree	22.6	17.71	26.4	0.169
Neonatal	Largest component (fraction)	0.620	0.574	0.690	0.305

Table Sb. Degree of selected hub proteins versus random protein sets. Degree of selected immune hub proteins in the observed networks compared with their degree in 5,000 random sets of equal size forced to contain the protein. P (BH), Benjamini-Hochberg adjusted P-value within each group. Values with adjusted P < 0.05 are in bold. IL6 was not present in either protein set and was therefore not tested; TNF and TLR4 were present in the maternal set only.

Network	Hub	Observed degree	Null mean	Null 95th pct	Empirical P	P (BH)
Maternal	TNF	77	68.54	79	0.0998	0.150
Maternal	TLR4	49	39.75	48	0.043	0.097
Maternal	CD40LG	40	28.17	35	0.0066	**0.026**
Maternal	CCL3	33	22.05	29	0.0060	**0.026**
Maternal	CCL4	24	19.97	26	0.172	0.221
Maternal	CXCL6	7	8.35	13	0.760	0.760
Maternal	CXCL8	44	36.55	45	0.082	0.147
Maternal	CXCL1	26	23.42	30	0.294	0.330
Maternal	CCL2	47	35.31	43	0.0088	**0.026**
Neonatal	CD40LG	23	16.22	22	0.036	0.146
Neonatal	CXCL8	23	21.69	28	0.403	0.430
Neonatal	CXCL6	7	6.23	10	0.430	0.430
Neonatal	CXCL1	20	14.54	20	0.073	0.146
Neonatal	CCL4	19	13.46	19	0.061	0.146
Neonatal	CCL28	5	2.87	6	0.150	0.200
Neonatal	CCL7	12	6.48	10	0.017	0.138
Neonatal	CCL26	9	5.88	10	0.123	0.197
